# Association of Preoperative Lactate and LDH Levels with Resection Length in Acute Mesenteric Ischemia

**DOI:** 10.3390/medicina62101997

**Published:** 2026-10-15

**Authors:** Mehmet Mert Hidiroglu, Ahmet Yavuz

**Affiliations:** Department of General Surgery, Gülhane Training and Research Hospital, University of Health Sciences, Ankara 06010, Türkiye; ayavuz.95@gmail.com

**Keywords:** acute mesenteric ischemia, lactate, lactate dehydrogenase, small bowel

## Abstract

*Background and Objectives:* This study examined the associations of admission lactate and lactate dehydrogenase (LDH) levels with total small bowel resection length in patients with acute mesenteric ischemia (AMI) who underwent resection. *Materials and Methods:* This retrospective, single-center study included 71 patients who underwent small bowel resection for AMI. Associations were assessed using Spearman’s correlation and linear regression adjusted for albumin and AMI etiology. Heteroscedasticity-consistent HC3 standard errors were used for regression inference. Model diagnostics and sensitivity analyses were performed. Admission biomarker levels were also compared by in-hospital mortality status in an exploratory analysis. *Results:* The median resection length was 90 cm (range, 20–360 cm) and this correlated with lactate (ρ = 0.551, *p* < 0.001) and LDH (ρ = 0.317, *p* = 0.007). In the etiology-adjusted model, lactate (B = 12.286, HC3 95% CI: 4.449–20.123; *p* = 0.003) and LDH (B = 0.035, HC3 95% CI: 0.014–0.056; *p* = 0.002) remained associated with resection length. The apparent R^2^ was 0.567, and both associations remained positive after influential observations were excluded. In exploratory unadjusted comparisons, non-survivors had higher admission lactate and LDH levels than survivors. *Conclusions:* Admission lactate and LDH levels were associated with resection length in this selected surgical cohort. Individual-level predictive accuracy was not established, and these biomarkers cannot currently guide operative decisions.

## 1. Introduction

Acute mesenteric ischemia (AMI) is a life-threatening condition characterized by an abrupt reduction in intestinal perfusion, which can lead to irreversible bowel damage and substantial mortality if not promptly managed [1]. The initially nonspecific clinical presentation of the disease complicates diagnosis, while delays in diagnosis and treatment may lead to the progression of intestinal injury and worsening clinical outcomes [2]. Despite advances in diagnostic imaging and treatment options, AMI continues to be associated with high mortality rates [3].

Although clinical evaluation and contrast-enhanced computed tomography angiography form the basis of the diagnostic approach to AMI, surgical exploration and resection are essential components of treatment in patients who develop necrosis [4]. The fundamental principle of surgical treatment is to resect necrotic segments while preserving viable or potentially salvageable bowel tissue. Because extensive small bowel resections may lead to serious long-term consequences, such as short bowel syndrome, limiting bowel loss is one of the principal goals of AMI management [4].

The consequences of extensive small bowel resection depend not only on the length removed but also on the remaining bowel length, preservation of the ileocecal valve, and colonic continuity. Substantial intestinal loss may compromise nutritional autonomy, potentially resulting in short bowel syndrome or intestinal failure and necessitating prolonged nutritional and rehabilitative care [5]. The extent of a bowel requiring resection is determined by several factors, including the distribution of ischemic injury, restoration of perfusion, and intraoperative assessment of viability. Although CT angiography provides essential information about vascular pathology and intestinal injury, the final resection margins are established during surgical treatment. These considerations have prompted investigations of whether routinely available preoperative measurements are associated with the amount of bowel ultimately resected.

Lactate and lactate dehydrogenase (LDH) are routinely available markers that may reflect tissue hypoperfusion and cellular injury, respectively. However, both are nonspecific and may be affected by conditions beyond intestinal ischemia. Previous investigations have not consistently demonstrated a significant relationship between preoperative lactate levels and the extent of intestinal ischemia, necrosis, or surgical resection, with some studies evaluating small bowel and colonic involvement together [6,7,8,9]. Current evidence also does not support using these markers alone to confirm or exclude AMI [4,10]. Whether admission lactate and LDH levels are associated with the measured length of resected small bowel in a cohort excluding concomitant colonic resection remains insufficiently characterized.

This study aimed to examine the associations of admission lactate and LDH levels with total small bowel resection length in patients who underwent resection for AMI. The analysis was restricted to this surgical subgroup and was intended to assess associations rather than develop or validate a model for predicting individual resection length.

## 2. Materials and Methods

### 2.1. Study Design and Patient Selection

We retrospectively reviewed patients treated with small bowel resection for AMI at a single institution between January 2018 and January 2026. The study protocol received approval from the Clinical Research Ethics Committee of Gülhane Training and Research Hospital, University of Health Sciences, Ankara, Türkiye (23 June 2026; approval no. 2026/226). All study procedures complied with the principles of the Declaration of Helsinki. Given the retrospective design, the committee waived the requirement for informed consent. Hospital electronic records, operative notes, radiological examinations, and pathology reports were reviewed, and all data were de-identified before analysis. The diagnosis of acute mesenteric ischemia was confirmed based on the combined assessment of clinical findings, contrast-enhanced abdominal computed tomography/computed tomography angiography, intraoperative evidence of intestinal ischemia or necrosis, and histopathological examination of the resected specimens.

Patients aged 18 years or older who underwent emergency surgical exploration for acute mesenteric ischemia were assessed for inclusion. Patients with chronic mesenteric ischemia and those who developed intestinal ischemia due to causes other than primary mesenteric vascular disease, such as strangulated hernia, adhesions, or volvulus, were excluded.

During the specified study period, a total of 112 patients who underwent emergency surgical exploration for acute mesenteric ischemia were assessed for eligibility. A total of 5 patients did not undergo resection because diffuse intestinal ischemia incompatible with survival were identified during surgical exploration, and 18 patients whose procedures included colon resection were excluded. A total of 10 patients in whom intestinal ischemia was identified but resection was not required because bowel viability improved during intraoperative assessment were also excluded. In addition, 8 patients with missing basic preoperative laboratory data and/or data on resection length were excluded. Consequently, 71 patients who underwent small bowel resection and in whom the total length of resected bowel could be reliably determined were included in the final analysis. Consecutive inclusion was applied to all patients meeting the eligibility criteria within the study period, without a prespecified sample size calculation. Figure 1 summarizes the selection process.

### 2.2. Data Collection and Definition of Variables

We collected information on age, sex, body mass index, smoking status, alcohol consumption, and previous abdominal operations. The underlying vascular etiology was classified using radiological and operative findings. Hospital stay was calculated from the admission date to discharge or death, and all-cause death occurring before discharge was recorded as in-hospital mortality.

Laboratory data were retrieved from the initial emergency department blood collection, obtained before fluid resuscitation, vasopressor administration, surgery, or other treatment. The recorded measurements comprised urea, creatinine, albumin, LDH, CRP, blood pH, lactate, leukocyte count, hemoglobin, platelet count, differential leukocyte counts, and RDW-SD. LDH levels were measured using a Beckman Coulter AU5800 clinical chemistry analyzer (Beckman Coulter, Brea, CA, USA), whereas lactate levels were measured using a Radiometer ABL800 blood gas analyzer (Radiometer Medical ApS, Brønshøj, Denmark). The interval from symptom onset to blood sampling could not be reliably determined from the retrospective records. Clinical and biochemical information were documented in a structured dataset independently of patient outcomes, followed by checks for data consistency before the statistical analyses.

### 2.3. Surgical Data and Outcome Measures

The surgical procedure performed was classified according to the operative reports as segmental small bowel resection with ostomy or segmental small bowel resection with anastomosis. The primary outcome variable was the total length of resected small bowel. The primary objective was to examine its associations with preoperative lactate and LDH levels. Associations with other laboratory parameters were assessed as exploratory analyses. Resection length was obtained primarily from the length documented intraoperatively in the operative report; when both operative and pathology measurements were available, the operative measurement took precedence. If an operative measurement was unavailable, the length documented in the pathology report was used, provided that the specimen had been measured before fixation. Whether the individuals who originally measured resection length were aware of the admission lactate and LDH results could not be established retrospectively. Total resection length included all of the small bowel removed during the index hospitalization, including bowel resected during a second-look or subsequent operation. Lengths of segments removed in separate operations were summed. The relationships of preoperative clinical and laboratory parameters with total resection length were evaluated. Death occurring during the index hospital admission was also evaluated as a secondary exploratory outcome.

### 2.4. Statistical Analysis

Data analysis was conducted using IBM SPSS Statistics version 27.0 (IBM Corp., Armonk, NY, USA), with Python version 3.13.5 and the statsmodels package version 0.14.6 used for HC3-robust regression inference. Continuous data distributions were examined using the Shapiro–Wilk test, graphical inspection of histograms, and normal Q–Q plots. Numerical variables were summarized by their median, range, and mean ± standard deviation, while frequencies and percentages were used for categorical data. Spearman’s rho (ρ) was calculated to quantify monotonic relationships between preoperative variables and resected bowel length.

Lactate and LDH were the primary biomarkers of interest; analyses of the remaining laboratory variables were exploratory. Associations with resected small bowel length were first examined using univariable linear regression. Model 1 included lactate, LDH, and albumin, selected on the basis of clinical relevance while limiting the number of covariates in this sample of 71 patients. Model 2 additionally adjusted for AMI etiology, entered as two indicator variables for arterial embolism and arterial thrombosis, with venous thrombosis as the reference category. Both models used the Enter method. The results were reported as unstandardized coefficients (B) with 95% confidence intervals (CIs) and standardized coefficients (β). Linearity and heteroscedasticity were assessed using residual-versus-fitted plots, and the distribution of residuals was examined using normal Q–Q plots. Because residual dispersion varied across fitted values, heteroscedasticity-consistent standard errors using the HC3 estimator were used for statistical inference in the linear regression analyses. Accordingly, the regression *p*-values and 95% CIs were based on HC3 robust standard errors, whereas the point estimates (B) were ordinary least-squares coefficients. Influential observations were screened using Cook’s distance, with values exceeding 4/*n* flagged for review. As a sensitivity analysis, Model 2 was refitted after excluding observations above this threshold; all observations remained in the primary analysis. To investigate the discrepancy between the Spearman correlation and the standardized univariable regression coefficient for LDH, the univariable LDH model was also refitted after excluding observations with Cook’s distance above 4/*n*. Coefficient stability was additionally investigated through 2000 bootstrap iterations in which patients were randomly drawn with replacement from the complete study cohort, rather than resampling regression residuals. Bias-corrected and accelerated (BCa) 95% confidence intervals were derived as a supplementary sensitivity analysis. Potential multicollinearity was examined using tolerance statistics and variance inflation factors (VIFs), while model fit was described by R^2^ and adjusted R^2^. In the exploratory mortality analysis, admission lactate, LDH, and albumin levels were compared between survivors and non-survivors using the Mann–Whitney U test. All statistical tests were two-sided, with significance defined at *p* < 0.05.

## 3. Results

The final analysis comprised 71 patients who underwent small bowel resection for AMI. The median age was 70 years (range, 42–97), and the cohort included 36 women (50.7%) and 35 men (49.3%). Venous thrombosis accounted for 42.3% of cases, arterial embolism for 38.0%, and arterial thrombosis for 19.7%. None of the included patients had non-occlusive mesenteric ischemia (NOMI). In-hospital mortality was 32.4%, while the median resection length was 90 cm (range, 20–360). Baseline demographic, clinical, and biochemical data are detailed in Table 1.

Spearman correlation analysis demonstrated positive associations of resected bowel length with both primary biomarkers: lactate (ρ = 0.551, *p* < 0.001) and LDH (ρ = 0.317, *p* = 0.007). Among the exploratory variables, CRP showed a positive correlation (ρ = 0.255, *p* = 0.032), whereas albumin (ρ = −0.277, *p* = 0.020) and pH (ρ = −0.236, *p* = 0.048) showed negative correlations. None of the remaining variables demonstrated a statistically significant correlation. These findings are presented in Table 2, with the lactate and LDH associations illustrated in Figure 2A and Figure 2B, respectively.

In univariable linear regression analyses, the primary biomarkers, lactate and LDH, were associated with resected bowel length. Using HC3 heteroscedasticity-robust inference, exploratory analyses also identified nominally significant associations for albumin, CRP, pH, and hemoglobin; these *p*-values were not adjusted for multiple testing (Table 3).

In the initial multivariable linear regression model, using HC3 heteroscedasticity-robust inference, admission lactate (B = 11.572, HC3 95% CI: 3.845–19.299; β = 0.403; *p* = 0.004) and LDH (B = 0.034, HC3 95% CI: 0.013–0.054; β = 0.340; *p* = 0.001) were associated with resected bowel length. Albumin was not significantly associated with resection length (*p* = 0.218). After adjustment for AMI etiology, the associations persisted for lactate (B = 12.286, HC3 95% CI: 4.449–20.123; β = 0.428; *p* = 0.003) and LDH (B = 0.035, HC3 95% CI: 0.014–0.056; β = 0.349; *p* = 0.002). Neither arterial embolism (*p* = 0.068) nor arterial thrombosis (*p* = 0.834) showed a statistically significant difference from venous thrombosis, the reference category (Table 4).

Diagnostic assessment of the etiology-adjusted model showed several large positive residuals in the residual-versus-fitted plot and deviations in the upper tail of the normal Q–Q plot (Figure 3). No clear curvilinear pattern was apparent, but residual dispersion varied across fitted values; therefore, homoscedasticity could not be established by visual inspection. Eight observations had Cook’s distance values above 4/*n* (approximately 0.056), with a maximum value of 0.175. After excluding these observations in a sensitivity analysis (*n* = 63), the associations remained positive for LDH (B = 0.036, HC3 95% CI: 0.019–0.052; *p* < 0.001) and lactate (B = 15.115, HC3 95% CI: 9.324–20.905; *p* < 0.001). All 71 patients were retained in the primary analysis. In the full etiology-adjusted model, 2000-resample BCa bootstrap 95% confidence intervals excluded zero for both LDH (0.012–0.053) and lactate (4.737–20.388).

To assess whether influential observations contributed to the difference between the Spearman correlation and the standardized univariable regression coefficient for LDH, the univariable analysis was repeated after excluding three observations with Cook’s distance above 4/*n*. The association persisted (B = 0.068, HC3 95% CI: 0.056–0.081; β = 0.582; *p* < 0.001) compared with B = 0.064 (HC3 95% CI: 0.054–0.074; β = 0.643; *p* < 0.001) in the full cohort.

In an exploratory analysis of in-hospital mortality, admission lactate and LDH levels were higher among non-survivors (*n* = 23) than survivors (*n* = 48). Median lactate levels were 5.8 versus 2.8 mmol/L (*p* < 0.001), and median LDH levels were 435 versus 278.5 U/L (*p* = 0.004), respectively. Median albumin levels were lower among non-survivors (2.9 versus 3.4 g/dL; *p* = 0.002) (Table 5).

## 4. Discussion

In this selected cohort of patients who underwent small bowel resection for acute mesenteric ischemia (AMI), admission lactate and LDH levels were associated with total resection length. Both associations persisted after adjustments for AMI etiology. These findings address a quantitative surgical outcome, but they do not establish that either biomarker can accurately estimate the length of bowel that an individual patient will require resection. Lactate and LDH should therefore be interpreted as markers associated with resection length in this cohort, rather than as validated tools for operative planning or counseling.

One of the main challenges in the surgical treatment of AMI is achieving a balance between adequate resection of irreversibly damaged bowel segments and maximal preservation of viable or salvageable intestinal tissue. The amount of bowel removed has potential consequences for subsequent intestinal function, although resection length alone does not capture the length or function of the remaining bowel. Extensive intestinal resection may substantially impair gastrointestinal function and nutritional independence, occasionally resulting in short bowel syndrome or chronic intestinal failure that necessitates prolonged parenteral support and specialized multidisciplinary care [11]. In a series of 212 surgically treated patients, the development of short bowel syndrome with dependence on parenteral nutrition in approximately one-quarter of the patients demonstrates the clinical consequences of extensive bowel loss among survivors [12]. Resection extent has also been examined in relation to early outcomes, including mortality [13]. Nevertheless, all patients with AMI require urgent clinical assessment; the present findings do not identify a subgroup for whom a different operative decision would be appropriate.

Clinical examination, imaging, and intraoperative assessment remain central to the evaluation of bowel viability. In addition to demonstrating mesenteric vascular pathology, CT angiography enables evaluation of findings accompanying intestinal injury, such as bowel wall perfusion, dilatation, pneumatosis, and portal venous gas [14]. Studies examining imaging findings associated with transmural necrosis have highlighted features such as pneumatosis intestinalis, advanced vascular obstruction, and decreased bowel wall enhancement [14,15,16]. However, the boundaries of viable bowel and the final extent of resection may change with intraoperative assessment, revascularization, and recovery of perfusion. A laboratory association with the length of bowel ultimately resected cannot substitute for these assessments.

Previous studies have differed in their patient selection, the intestinal segments evaluated, and whether the outcome was the extent of ischemia, necrosis, or surgical resection. These differences complicate direct comparisons with our results. Ambe et al. evaluated cases of small bowel, colonic, and multivisceral ischemia in the same analysis; categorized the length of ischemia; and found no significant relationship between lactate levels and the length of ischemia [7]. Monroy et al. evaluated small bowel and colonic necrosis using a common length measure and found no significant correlation between admission lactate and the length of necrosis [9]. Studer et al. examined serial preoperative lactate measurements and reported limited value for estimating the length of necrotic bowel [6]. A more recent surgical series including different intestinal segments also did not demonstrate a significant relationship between preoperative lactate and resection length [8]. By focusing exclusively on the total length of resected small bowel and excluding patients who underwent concomitant colonic resection, our study evaluated a more anatomically uniform surgical outcome than several previous investigations. This methodological distinction may have reduced outcome heterogeneity and contributed to the observed positive associations, although its independent effect cannot be established and the resulting restriction limits the generalizability of our findings.

The available clinical evidence regarding LDH is more limited. Although experimental models of mesenteric ischemia have shown that LDH levels increase after ischemia and that histopathological injury progresses with the duration of ischemia, the relationship between LDH and the amount of bowel resected has not been adequately defined in human studies [17]. In the prospective multicenter DIAGOMI study, LDH levels were higher in patients with intestinal necrosis; however, routine laboratory parameters alone could not reliably distinguish the presence and extent of necrosis [18]. In the series of 149 patients with AMI reported by Paladino et al., serum LDH levels were higher among patients who died in hospitals; however, LDH levels were not significantly associated with the extent categories of intestinal ischemia [19]. Our findings add an observed association between admission LDH and small bowel resection length without establishing that LDH specifically reflects the amount of ischemic bowel.

The associations are biologically plausible, but their mechanisms cannot be determined from these data. Lactate may reflect systemic hypoperfusion and altered metabolism, while LDH may increase with cellular injury from several sources; neither is specific to the small bowel. In the etiology-adjusted model, a 1 mmol/L higher lactate level was associated with approximately 12.3 cm greater resection length, whereas a 100 U/L higher LDH level was associated with approximately 3.5 cm greater resection length, conditional on the other model variables. These coefficients describe average associations within the analyzed cohort. They should not be applied to estimate an individual patient’s bowel loss. The apparent R^2^ of 0.567 describes fit within the study cohort. Because the model was not validated and individual-level prediction errors were not assessed, this value is not evidence of clinical predictive accuracy.

The diagnostic and sensitivity analyses also qualify the regression findings. The residual Q–Q plot deviated in its upper tail, residual dispersion varied across fitted values, and eight observations exceeded the Cook’s distance screening threshold. The associations of lactate and LDH remained statistically significant when heteroscedasticity-consistent HC3 standard errors were used for inference. The associations with lactate and LDH remained positive after these observations were excluded, and their bootstrap confidence intervals excluded zero in the full etiology-adjusted model. Nevertheless, these checks do not remove uncertainty arising from influential observations, model assumptions, or unmeasured confounding. The difference between LDH’s Spearman correlation and standardized univariable regression coefficient further cautions against interpreting a single coefficient as a measure of clinical utility.

Venous thrombosis was the largest individual etiology category in the included cohort (42.3%), whereas arterial embolism and arterial thrombosis together accounted for 57.7%. No cases of NOMI were identified among the 71 analyzed patients; this observation does not establish the absence of NOMI among patients excluded from the analysis. Consequently, the present findings cannot be generalized to patients with NOMI. The etiology distribution among the 71 analyzed patients should not be taken as the distribution among all patients with AMI. In particular, eligibility depended on undergoing a measurable small bowel resection; patients whose bowel remained viable and patients with diffuse ischemia for which resection was not performed were excluded. The direction and magnitude of any resulting selection bias cannot be determined from the available data. Adjustments for the three recorded etiologies did not remove the observed lactate and LDH associations, but they cannot account for differences in disease stage or other unmeasured clinical factors.

In the additional exploratory mortality analysis, non-survivors had higher admission lactate and LDH levels and lower albumin levels than survivors. This finding addresses a clinically relevant question, but the comparisons were unadjusted and were performed within the same selected resection cohort. They do not establish independent associations with mortality, define useful thresholds, or demonstrate mortality prediction. This study also cannot evaluate whether these markers identify a need for laparotomy or safely exclude bowel necrosis, because patients who did not undergo small bowel resection were not included in the analysis.

The findings should be considered in light of several methodological constraints. First, the analysis was based on a retrospective dataset from a single institution involving 71 patients, which limits the precision of the estimates and their applicability to other clinical populations. Restriction to patients who underwent small bowel resection excluded ten patients whose bowel did not require resection after intraoperative assessment and five patients with diffuse ischemia incompatible with survival who did not undergo resection. The latter may have included some of the most severe presentations, while patients with viable bowel could also have elevated lactate or LDH. Patients whose procedures included colon resection were excluded, and the characteristics of eight patients excluded because of missing data could not be reliably compared with those of the included patients. Together, these exclusions create substantial potential for selection bias.

Although the laboratory values were obtained from the first emergency department’s blood samples, the interval from symptom onset to sampling could not be reliably determined. Patients may therefore have been sampled at different stages of disease. Hemodynamic status, organ dysfunction, time to surgery, revascularization, and other clinical factors could not be fully incorporated into the analysis. Consequently, residual confounding remains possible despite adjustment for AMI etiology, and the observed associations should not be interpreted as independent causal effects of lactate or LDH on resection extent. Potential hemolysis during blood collection or handling may also have affected LDH measurements, but its contribution could not be assessed retrospectively. Moreover, total resection length reflects not only the extent of ischemic injury but also the surgeon’s assessment of bowel viability, decisions regarding revascularization, recovery of bowel perfusion during surgery, and institutional practice concerning reassessment and second-look operations. Operative measurements may be imprecise, and pathology measurements, when used, may differ from measurements made in situ despite being obtained before fixation.

Finally, laboratory analyses beyond the primary lactate and LDH questions, including the mortality comparisons, were exploratory, and no adjustment was made for multiple testing. Some nominally significant findings may therefore be false positives. No clinically useful thresholds, prediction intervals, individual-level accuracy estimates, or model validation were available. At present, the observed associations cannot guide operative counseling, determine whether resection is required, or change decisions about laparotomy. Their clinical relevance requires assessment in larger cohorts that include the broader spectrum of patients with AMI.

## 5. Conclusions

In this selected cohort of patients who underwent small bowel resection for acute mesenteric ischemia, higher admission lactate and LDH levels were associated with greater total resection length. These associations persisted after adjustment for AMI etiology and in sensitivity analyses. However, the findings do not establish individual-level predictive accuracy and cannot currently guide operative counseling or decisions about resection. Studies including the broader spectrum of patients with AMI are needed to assess whether these associations have clinical value.

## Figures and Tables

**Figure 1:**
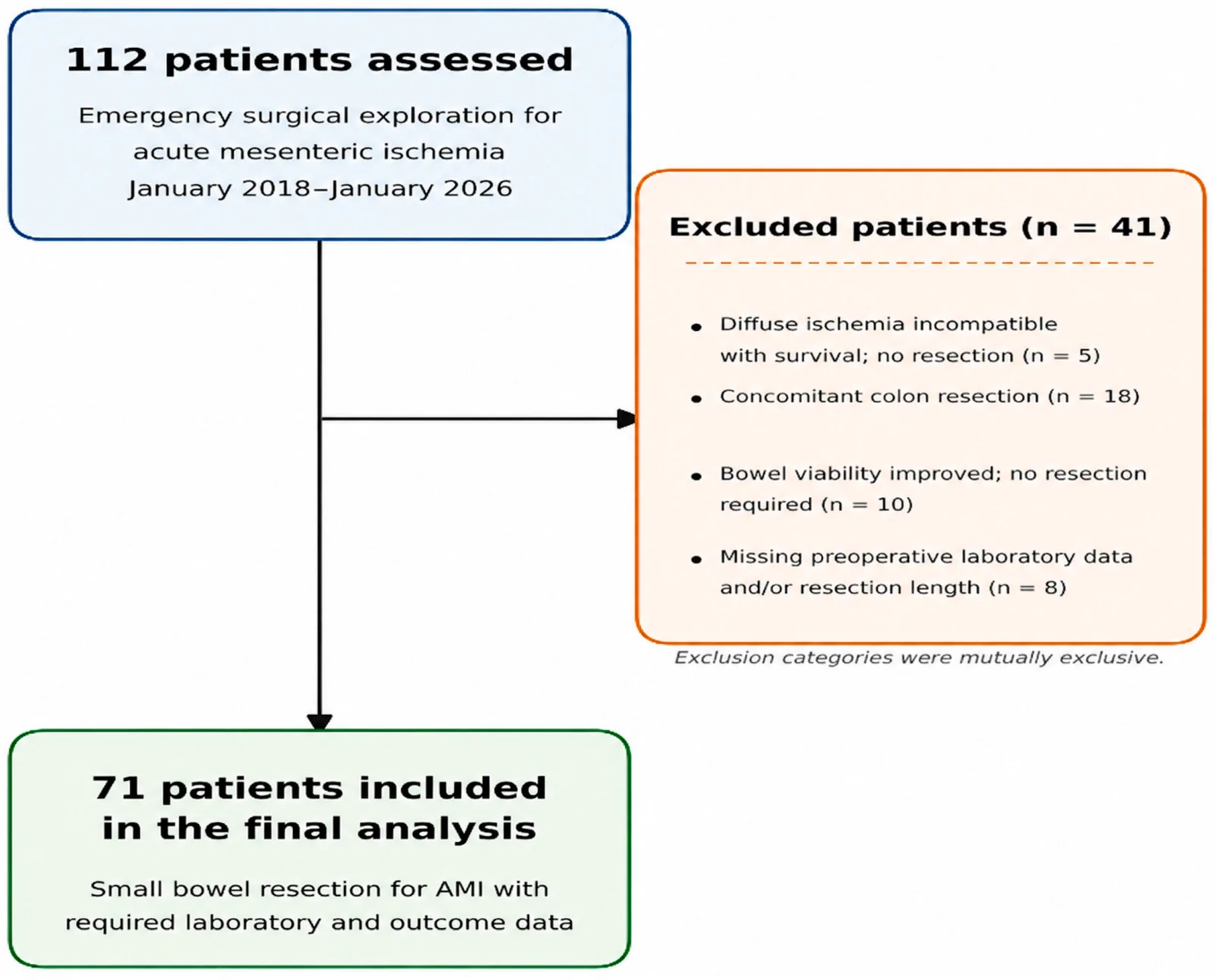
Patient selection flow diagram. Exclusion categories were mutually exclusive. AMI, acute mesenteric ischemia.

**Figure 2:**
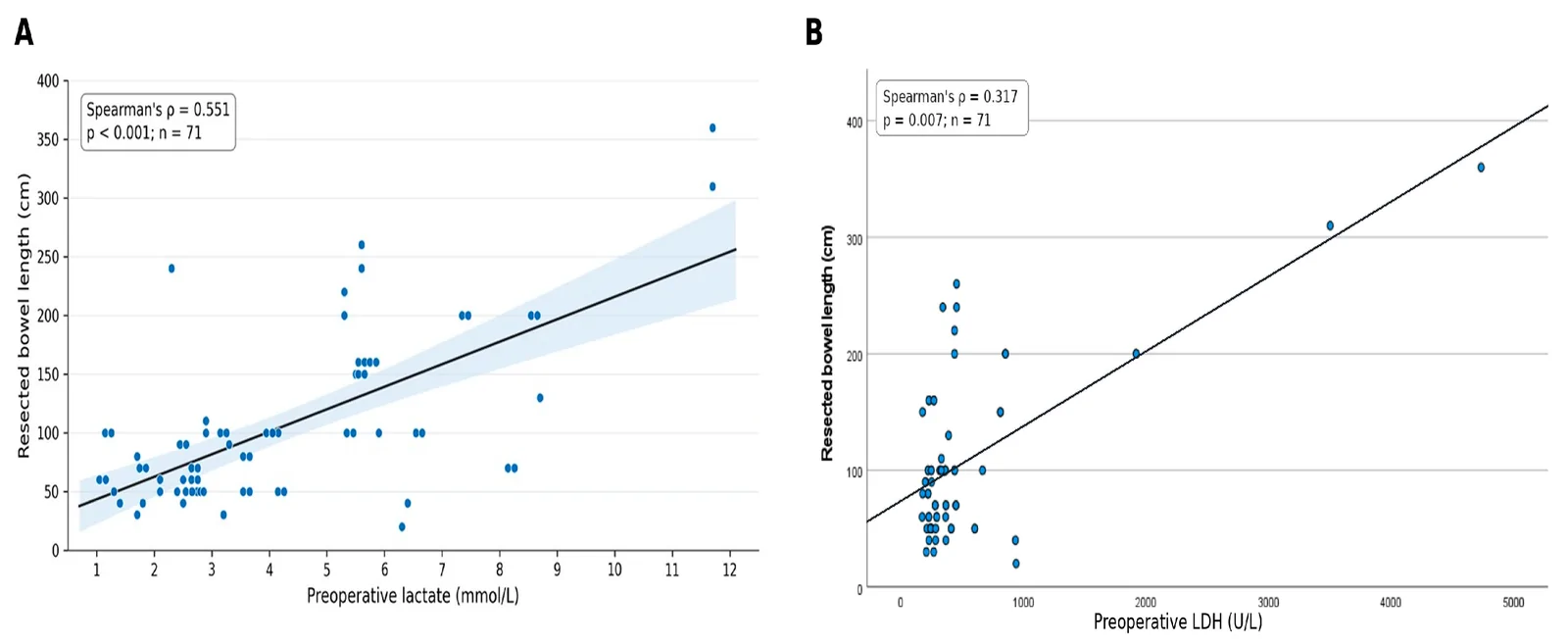
Associations of preoperative lactate and LDH levels with resected bowel length. (**A**) Lactate and (**B**) LDH. Each point represents one patient. The solid lines represent univariable linear regression fits; the shaded area in panel (**A**) represents the confidence interval around the fitted line. The reported Spearman correlation coefficients were calculated separately from these fitted lines.

**Figure 3:**
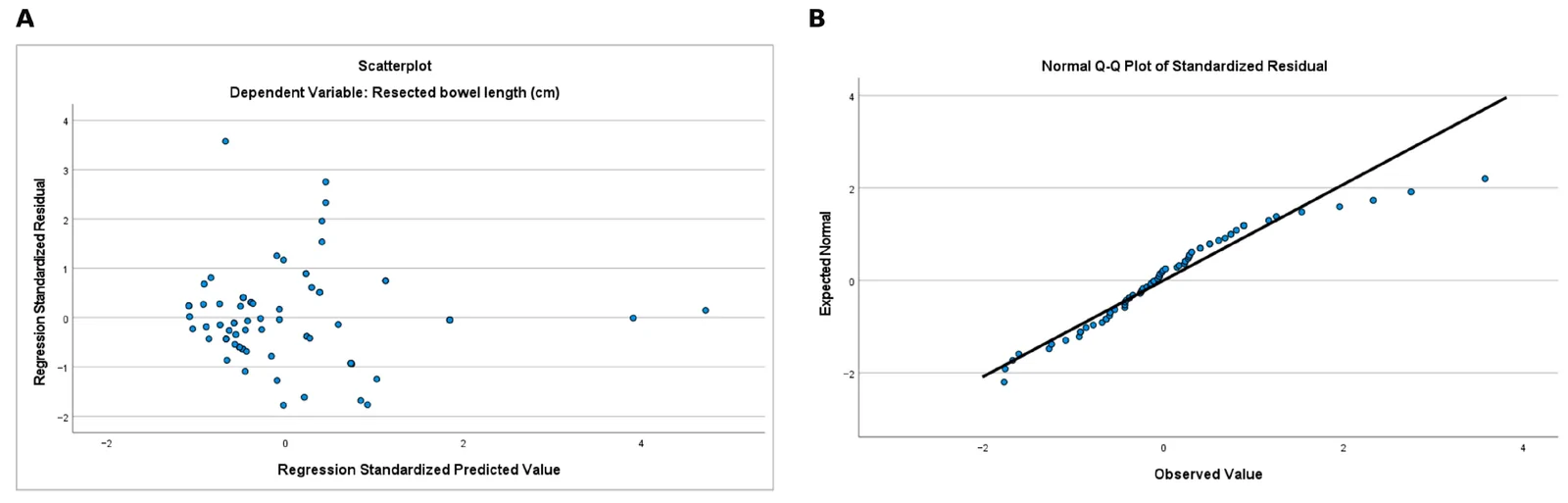
Diagnostic plots for the etiology-adjusted multivariable linear regression model: (**A**) standardized residuals versus standardized predicted values; (**B**) normal Q–Q plot of standardized residuals.

**Table 1:** Baseline characteristics and laboratory findings of the study population.

**Parameters**	**Values**
Age (years)	70 (42–97), 70.04 ± 13.97
Sex	
Female	36 (50.7%)
Male	35 (49.3%)
Smoking	
No	23 (32.4%)
Yes	48 (67.6%)
Alcohol consumption	
No	54 (76.1%)
Yes	17 (23.9%)
BMI (kg/m^2^)	29 (20–36), 29.27 ± 3.77
Previous abdominal surgery	
No	38 (53.5%)
Yes	33 (46.5%)
Etiology	
Arterial embolism	27 (38%)
Arterial thrombosis	14 (19.7%)
Venous thrombosis	30 (42.3%)
In-hospital mortality	
No	48 (67.6%)
Yes	23 (32.4%)
Length of hospital stay (days)	8 (1–70), 8.97 ± 11.03
Urea (mg/dL)	66 (12–242), 79.89 ± 53.07
Creatinine (mg/dL)	1.35 (0.38–7.68), 1.63 ± 1.05
Albumin (g/dL)	3.2 (1.8–5.7), 3.27 ± 0.79
LDH (U/L)	316 (171–4732), 512.18 ± 702.62
CRP (mg/L)	152 (7–490), 183.62 ± 139.06
pH	7.38 (7.12–7.57), 7.36 ± 0.09
Lactate (mmol/L)	3.6 (1.1–11.7), 4.24 ± 2.42
WBC (×10^9^/L)	14.1 (2.1–34.4), 16 ± 7.42
Hb (g/dL)	13.4 (6.6–19.3), 13.33 ± 2.9
PLT (×10^9^/L)	246 (109–810), 273.52 ± 125.01
Neutrophils (×10^9^/L)	12.6 (1.4–31), 13.24 ± 7.05
Lymphocytes (×10^9^/L)	1.15 (0.2–4.4), 1.29 ± 0.87
Monocytes (×10^9^/L)	0.8 (0–2.6), 0.91 ± 0.55
RDW-SD (fL)	48.9 (36.8–68.7), 49.18 ± 6.99
Surgical procedure	
Segmental small bowel resection + ostomy	15 (21.1%)
Segmental small bowel resection + anastomosis	56 (78.9%)
Total length of resected small bowel (cm)	90 (20–360), 105.49 ± 69.56

Continuous variables are presented as median (minimum–maximum) and mean ± standard deviation; categorical variables are presented as *n* (%).

**Table 2:** Spearman’s correlation analysis between preoperative clinical and laboratory variables and the length of resected bowel.

**Variable**	**Spearman’s Correlation Coefficient**	***p*** **-Value**
Age (years)	0.159	0.184
BMI (kg/m^2^)	−0.038	0.753
Urea (mg/dL)	0.157	0.191
Creatinine (mg/dL)	0.027	0.826
Albumin (g/dL)	−0.277	0.020
LDH (U/L)	0.317	0.007
CRP (mg/L)	0.255	0.032
pH	−0.236	0.048
Lactate (mmol/L)	0.551	<0.001
WBC (×10^9^/L)	0.212	0.076
Hb (g/dL)	−0.229	0.055
PLT (×10^9^/L)	0.100	0.409
Neutrophils (×10^9^/L)	0.196	0.102
Lymphocytes (×10^9^/L)	−0.221	0.064
Monocytes (×10^9^/L)	0.148	0.219
RDW-SD (fL)	0.191	0.110

**Table 3:** Univariable linear regression analyses of variables associated with resected bowel length.

**Variables**	**B (HC3 95% CI)**	**β**	**HC3 *p*-Value**
Age (years)	0.908 (−0.295 to 2.111)	0.182	0.137
BMI (kg/m^2^)	−0.205 (−6.079 to 5.668)	−0.011	0.945
Urea (mg/dL)	0.046 (−0.248 to 0.341)	0.035	0.754
Creatinine (mg/dL)	−3.495 (−14.693 to 7.702)	−0.053	0.536
Albumin (g/dL)	−31.539 (−53.207 to −9.871)	−0.360	0.005
LDH (U/L)	0.064 (0.054 to 0.074)	0.643	<0.001
CRP (mg/L)	0.145 (0.007 to 0.282)	0.289	0.039
pH	−290.971 (−517.290 to −64.651)	−0.410	0.013
Lactate (mmol/L)	19.170 (11.881 to 26.459)	0.667	<0.001
WBC (×10^9^/L)	0.081 (−2.894 to 3.056)	0.009	0.957
Hb (g/dL)	−8.240 (−15.553 to −0.927)	−0.344	0.028
PLT (×10^9^/L)	0.018 (−0.105 to 0.140)	0.032	0.775
Neutrophils (×10^9^/L)	0.438 (−2.447 to 3.324)	0.044	0.763
Lymphocytes (×10^9^/L)	−17.439 (−35.176 to 0.299)	−0.219	0.054
Monocytes (×10^9^/L)	4.922 (−35.146 to 44.990)	0.039	0.807
RDW-SD (fL)	2.679 (−0.991 to 6.349)	0.269	0.150

**Table 4:** Multivariable linear regression analyses of variables associated with resected bowel length.

**Variables**	**B**	**β**	**HC3 95% CI**	**HC3 *p*-Value**	**Tolerance**	**VIF**
Model 1						
Albumin (g/dL)	−12.811	−0.146	−33.364 to 7.741	0.218	0.900	1.112
LDH (U/L)	0.034	0.340	0.013 to 0.054	0.001	0.566	1.765
Lactate (mmol/L)	11.572	0.403	3.845 to 19.299	0.004	0.562	1.780
Model 2						
Albumin (g/dL)	−12.714	−0.145	−32.402 to 6.975	0.202	0.864	1.158
LDH (U/L)	0.035	0.349	0.014 to 0.056	0.002	0.550	1.818
Lactate (mmol/L)	12.286	0.428	4.449 to 20.123	0.003	0.538	1.860
Arterial embolism	24.801	0.174	−1.887 to 51.490	0.068	0.841	1.188
Arterial thrombosis	3.996	0.023	−33.831 to 41.822	0.834	0.684	1.462

Model 1 included albumin, LDH, and lactate (R^2^ = 0.540; adjusted R^2^ = 0.520). Model 2 additionally adjusted for AMI etiology (R^2^ = 0.567; adjusted R^2^ = 0.534; Durbin–Watson statistic = 1.332). Venous thrombosis was the reference category for etiology. B coefficients for arterial embolism and arterial thrombosis represent differences in resection length relative to venous thrombosis, conditional on the other variables in Model 2.

**Table 5:** Exploratory comparison of admission laboratory parameters by in-hospital mortality status.

**Parameters**	**Survivors (*n*:48)**	**Non-Survivors (*n*:23)**	***p*** **-Value**
Albumin (g/dL)	3.4 (2.4–5.7), 3.49 ± 0.70	2.9 (1.8–4.3), 2.81 ± 0.78	0.002
LDH (U/L)	278.5 (171–937), 343.71 ± 188.18	435 (226–4732), 863.78 ± 1141.91	0.004
Lactate (mmol/L)	2.8 (1.1–8.7), 3.34 ± 1.75	5.8 (1.2–11.7), 6.12 ± 2.58	<0.001

Descriptive values are reported as median (range) and mean ± standard deviation. Between-group *p*-values were obtained from the Mann–Whitney U test.

## Data Availability

The datasets generated and/or analyzed during the current study are available from the corresponding author upon reasonable request. The data are not publicly available due to privacy and ethical restrictions.

## Abbreviations

The following abbreviations are used in this manuscript:

AMI	Acute mesenteric ischemia
BMI	Body mass index
CRP	C-reactive protein
CT	Computed tomography
CTA	Computed tomography angiography
Hb	Hemoglobin
LDH	Lactate dehydrogenase
PLT	Platelet count
RDW-SD	Red cell distribution width–standard deviation
WBC	White blood cell count
